# Oxidative Stress and Digestive Enzyme Activity of Flatfish Larvae in a Changing Ocean

**DOI:** 10.1371/journal.pone.0134082

**Published:** 2015-07-29

**Authors:** Marta S. Pimentel, Filipa Faleiro, Mário Diniz, Jorge Machado, Pedro Pousão-Ferreira, Myron A. Peck, Hans O. Pörtner, Rui Rosa

**Affiliations:** 1 MARE - Marine and Environmental Sciences Centre, Laboratório Marítimo da Guia, Faculdade de Ciências da Universidade de Lisboa, Av. Nossa Senhora do Cabo 939, 2750-374, Cascais, Portugal; 2 Instituto Ciências Biomédicas Abel Salazar, Universidade do Porto, Largo Prof. Abel Salazar 2, 4099-003, Porto, Portugal; 3 REQUIMTE, Departamento de Química, Centro de Química Fina e Biotecnologia, Faculdade de Ciências e Tecnologia, Universidade Nova de Lisboa, Quinta da Torre, 2829-516, Caparica, Portugal; 4 Instituto Português do Mar e da Atmosfera, Av. 5 de Outubro, 8700-305, Olhão, Portugal; 5 Institute for Hydrobiology and Fisheries Science, University of Hamburg, Olbersweg 24, 22767, Hamburg, Germany; 6 Alfred Wegener Institute for Polar and Marine Research, Animal Ecophysiology, Postfach 120161, 27515, Bremerhaven, Germany; Institute of Hydrobiology, Chinese Academy of Sciences, CHINA

## Abstract

Until now, it is not known how the antioxidant and digestive enzymatic machinery of fish early life stages will change with the combined effects of future ocean acidification and warming. Here we show that high *p*CO_2_ (~1600 μatm) significantly decreased metabolic rates (up to 27.4 %) of flatfish larvae, *Solea senegalensis*, at both present (18 °C) and warmer temperatures (+4 °C). Moreover, both warming and hypercapnia increased the heat shock response and the activity of antioxidant enzymes, namely catalase (CAT) and glutathione S-transferase (GST), mainly in post-metamorphic larvae (30 dph). The lack of changes in the activity of CAT and GST of pre-metamorphic larvae (10 dph) seems to indicate that earlier stages lack a fully-developed antioxidant defense system. Nevertheless, the heat shock and antioxidant responses of post-metamorphic larvae were not enough to avoid the peroxidative damage, which was greatly increased under future environmental conditions. Digestive enzymatic activity of *S*. *senegalensis* larvae was also affected by future predictions. Hypercapnic conditions led to a decrease in the activity of digestive enzymes, both pancreatic (up to 26.1 % for trypsin and 74.5 % for amylase) and intestinal enzymes (up to 36.1 % for alkaline phosphatase) in post-metamorphic larvae. Moreover, the impact of ocean acidification and warming on some of these physiological and biochemical variables (namely, lower OCR and higher HSP and MDA levels) were translated into larvae performance, being significantly correlated with decreased larval growth and survival or increased incidence of skeletal deformities. The increased vulnerability of flatfish early life stages under future ocean conditions is expected to potentially determine recruitment and population dynamics in marine ecosystems.

## Introduction

Ocean acidification and warming are among the most relevant environmental challenges that marine organisms will face in tomorrow’s oceans [1–4]. The continuous absorption of atmospheric CO_2_ by the oceans is expected to change seawater chemistry, with forecasts estimating a drop of 0.3–0.4 units in ocean pH by the year 2100. At the same time, the oceans are becoming warmer, and will continue as global surface temperature is expected to increase 1.1–6.4°C by the end of the century [5]. These environmental stressors may drive organisms outside their tolerance boundaries, compromising the overall fitness and survival of local populations.

Many organisms may cope with such climate-related changes, within limits, by adjusting mechanisms across levels of biological organization [4], including physiological protective mechanisms such as integrated heat shock and oxidative stress responses. When exposed to environmental fluctuations, organisms may be induced to produce heat shock proteins (HSP) to repair, refold, and eliminate damaged or denatured proteins [6]. Additionally, environmental stress may also induce the production of reactive oxygen species (ROS) [7]. The increase in ROS production may affect cellular integrity [8], and can injure cellular mechanisms by lipid peroxidation, one of the most frequent cellular injury processes where ROS react with membrane-associated lipids [7]. ROS production in marine organisms is controlled by efficient antioxidant capacity, characterized by a set of antioxidant enzymes which can together detoxify ROS [9].

When the above-mentioned protective mechanisms fail after exposure to environmental stress, organisms might limit the energy available, and growth, motility, ingestion, and digestion may suffer several functional disturbances [10]. In what concerns digestion, a correct maturation of the digestive system is essential to transform macronutrients from food into a form that can be easily digested, absorbed and assimilated, in order to supply dietary nutrients required for normal growth and development [11]. The digestive enzymes (pancreatic and brush border intestinal enzymes) are part of the metabolic regulatory mechanisms [10] and are thus widely used in studies as markers of fish larval development and as indicators of fish condition and physiological state [11–14]. The normal maturation of the enterocytes in developing fish larvae is characterized by a decrease of pancreatic enzyme activity (namely, trypsin and amylase), and by a marked increase in intestinal brush border membrane enzyme activity (such as alkaline phosphatase—ALP). This efficient brush border membrane digestion is representative of an adult mode of digestion [15]. A correlation between the major landmark events in digestive tract differentiation and the ontogenetic development of the digestive enzyme activities has been described in several fish species [16–19].

The activity of digestive enzymes is expected to be affected by external factors that modify metabolic functions, such as temperature and pH [10]. So far, the influence of ocean acidification on the digestive efficiency and enzymatic activity of marine organisms has been studied on marine invertebrate organisms [20–22]. The susceptibility of fish species to ocean acidification has received far less attention, since fish have developed an effective acid-base regulatory mechanism [23–25]. Nevertheless, the early life stages are expected to be more susceptible to changes in seawater *p*CO_2_ and more prone to extracellular changes than juvenile and adult fish [24,26]. Indeed, several morphological, physiological and behavioral disturbances have been observed in fish early stages [26–34], including the target species of this study, the flatfish *Solea senegalensis*. In a previous study, the survival, growth and development of sole larvae showed to be negatively impacted by ocean warming and acidification [see 29], but the underlying mechanisms remain unknown.

Here we provide a comprehensive set of physiological and biochemical responses of *S*. *senegalensis* early life stages to ocean warming (+4°C) and acidification (ΔpH = 0.5), which includes: i) oxygen consumption rates (OCR), ii) heat shock response (HSR; namely HSP70), iii) antioxidant enzyme activities (GST—glutathione S-transferase, and CAT—catalase), iv) lipid peroxidation (MDA—malondialdehyde concentration), and v) digestive enzymatic activities (trypsin, amylase and ALP). Additionally, a correlation analysis was performed to link these parameters with the morphological data from our previous work [29].

## Materials and Methods

### Ethics statement

This study was authorized by the Portuguese National Authority for Animal Health (Direcção-Geral de Alimentação e Veterinária), and it was performed in strict accordance with the recommendations of the Animal Care and Use Committee of the Faculty of Sciences of the University of Lisbon.

### Egg collection and larval rearing

Eggs of Senegal sole were collected from broodstock fish at IPMA—Estação Piloto de Piscicultura de Olhão (CRIP Sul, Olhão, Portugal) in June 2012, and transferred to the aquaculture facilities in Laboratório Marítimo da Guia (Cascais, Portugal). Senegal sole larvae were reared and collected in the same experiment published by Pimentel *et al*. [29].

After a short (2 h) acclimation period, eggs and larvae were exposed for one month to: i) 18°C—the mean sea surface temperature in summer (sSST) and normocapnia (*p*CO_2_ = ~400 μatm), ii) 18°C and hypercapnia (*p*CO_2_ = ~1600 μatm; ΔpH = 0.5), iii) 22°C—the future sSST warming scenario for the western coast of Portugal in 2100 (+ 4°C) and normocapnia, and iv) 22°C and hypercapnia. This species inhabits the Western Iberian Upwelling Ecosystem, part of the Canary Current Upwelling System, one of the four major eastern boundary currents of the world. In these regions, actual *p*CO_2_ levels may reach up to 500 μatm [35–37] and are thus expected to exceed the level of 1000 μatm projected for 2100 [5].

Larvae were reared in twelve recirculating seawater systems (three per treatment). Newly-hatched larvae were distributed randomly into three 19-L rearing tanks at a density of 70 larvae L^-1^. Feeding was adjusted according to larval development at each experimental condition. Larvae opened the mouth around 2 dph and started to feed on rotifers, *Brachionus plicatilis*. Enriched (AlgaMac-3050) *Artemia* metanauplii were introduced at 5 dph and their proportion in the diet was gradually increased, becoming the only prey offered at 8 dph. After larval settlement, frozen metanauplii were also introduced in the tank. Rotifer and *Artemia* density were adjusted twice a day to assure optimal prey density.

Temperatures (18.0 ± 0.2 and 22.0 ± 0.2°C) were controlled via Heilea chillers (Guangdong, China). The pH was automatically adjusted in each tank via a Profilux (Kaiserslautern, Germany) connected to a pH probe (WaterTech pH 201S) and operating a solenoid valve connected to a CO_2_ tank. The pH of each tank was also measured daily using a portable pH meter (SevenGo pro SG8, Mettler Toledo), in order to cross-calibrate the pH probes and to adjust the set points of the systems as required. Average pH values of the control and low pH treatments were 8.02 ± 0.05 and 7.51 ± 0.05, respectively. The salinity was kept at 35.4 ± 0.4. Ammonia and nitrite were monitored regularly and maintained within recommended levels.

Seawater carbonate system speciation (see S1 Table) was calculated weekly from total alkalinity (determined according to Sarazin *et al*. [38]) and pH measurements. Total dissolved inorganic carbon (C_T_), *p*CO_2_, bicarbonate concentration and aragonite saturation were calculated using the CO2SYS software [39], with dissociation constants from Mehrbach *et al*. [40] as refitted by Dickson & Millero [41].

Fish larvae were collected at 10 dph (pre-metamorphic stage), 20 dph (intermediate stage—undergoing metamorphosis) and 30 dph (post-metamorphic stage). Larvae were immediately placed in liquid nitrogen and then stored at -80°C for posterior biochemical analyses.

### Oxygen consumption rates

Oxygen consumption measurements were determined according to previously established methods [42,43]. Nine pre-metamorphic (10 dph) and nine post-metamorphic (30 dph) larvae from each treatment (three per replicate) were individually placed in sealed water-jacketed respirometry chambers (RC300 Respiration cell, Strathkelvin, North Lanarkshire, Scotland) containing 1-μm filtered and UV-irradiated seawater from each treatment condition mixed with antibiotics (50 mg L^-1^ streptomycin) to avoid bacterial respiration. Water volumes were adjusted in relation to animal mass (up to 10 mL) to minimize larval stress. Respiration chambers were immersed in water baths (Lauda, Lauda-Königshofen, Germany) to control temperature. The respiratory runs occurred after an acclimation period of about 2 h and lasted between 3 to 6 h. Oxygen consumption was also measured in chambers containing just water (blanks) for correction of possible bacterial respiratory activity. Oxygen concentrations were recorded with Clark-type O_2_ electrodes connected to a multi-channel oxygen interface (Model 928, Strathkelvin, North Lanarkshire, Scotland). At the end of the respirometry trials, the mean minimum level of oxygen achieved was of 86.8 ± 6.6%.

### Heat shock response, antioxidant enzymes and lipid peroxidation

#### Preparation of tissue extracts

After 10 and 30 days of acclimation to the different climate change scenarios, whole larvae were pooled from each replicate tank, comprising a total of three replicates per treatment. Homogenates were prepared using 150 mg wet tissue from each replicate tank. All samples were homogenized in 250 μL of phosphate buffered saline solution (PBS, pH 7.3, composed by 0.14 M NaCl, 2.7 mM KCl, 8.1 mM Na_2_HP0_4_ and 1.47 mM KH_2_P0_4_), by using a glass/PTFE Potter Elvehjem tissue grinder (Kartell, Italy). All homogenates were then centrifuged during 20 min at 14000 g at 4°C. HSP, antioxidant enzyme activities, lipid peroxidation and total protein expression were measured in the supernatant fraction. All enzyme assays were tested with commercial enzymes obtained from Sigma (Missouri, USA), and each sample was run in triplicate. The enzyme results were normalized by measuring the total protein content of the samples according to the Bradford method [44].

#### Heat shock response

HSP70 content (HSC70/HSP70) was assessed by ELISA (Enzyme-Linked Immunoabsorbent Assay) as previously described by Rosa *et al*. [43]. Briefly, a total of 5 μL of homogenate supernatant was diluted in 250 μL of PBS, and 50 μL of the diluted sample was added to 96-well microplates MICROLON600 (Greiner Bio-One GmbH, Germany) and incubated overnight at 4°C. Microplates were washed on the next day in 0.05% PBS-Tween-20 and 100 μL of blocking solution (1% Bovine Serum Albumin, BSA) was added to each well. For 2 hours, the microplates were incubated at room temperature in darkness. Then, 50 μL of a solution of 5 μg mL^-1^ primary antibody anti-HSP70/HSC70 (that detects both 72 and 73 kDa proteins, which corresponds to the molecular mass of inducible HSP70 and constitutive HSC70, respectively) was added to each well. Wells were then incubated at 37°C for 90 min. The non-linked antibodies were removed by washing the microplates, which were then incubated for 90 min at 37°C with 50 μL of the secondary antibody [anti-mouse IgG Fab specific, ALP conjugate (1 μg mL^-1^) from Sigma-Aldrich (Germany)]. After another wash, 100 μL of substrate p-nitrophenyl phosphate tablets (Sigma-Aldrich, Germany) was added to each well and incubated at room temperature (10 to 30 min). Subsequently, 50 μL of stop solution (3 M NaOH) was added to each well, and the absorbance was read at 405 nm in a 96-well microplate reader (BIO-RAD, Benchmark, USA). The amount of HSP70/HSC70 in the samples was then calculated from a standard curve of absorbance based on serial dilutions (from 0 to 2000 ng mL^-1^) of purified HSP70 active protein (Acris, USA). The results were expressed in relation to the protein content of the samples (ng HSP70/HSC70 mg protein^-1^).

#### Antioxidant enzymes

Glutathione S-transferase: GST activity was determined according to the procedure described by Rosa *et al*. [45] and Lopes *et al*. [46], optimized for a 96-well microplate. This assay uses 1-chloro-2,4-dinitrobenzene (CDNB) as substrate, which conjugates with the thiol group of the glutathione (GSH), causing an increase in absorbance. 180 μL of substrate solution (composed by 200 mM L-glutathione reduced, Dulbecco's PBS and 100 mM CDNB solution) was added to each well of a 96-well Nunclon microplate (Thermo Scientific Nunc, USA), along with 20 μL of GST standard or sample. Equine liver GST was used as a positive control to validate the assay. The enzyme activity was determined spectrophotometrically at 340 nm by measuring the formation of the conjugate of GSH and CDNB. The absorbance was recorded every minute for 6 min, using a plate reader (BioRad, California, USA). The increase in absorbance per minute was estimated and the reaction rate at 340 nm was determined using the CDNB extinction coefficient of 0.0053 ƐμM (μM^−1^ cm^−1^) as follows:

$$\text{GST activity }=\;\frac{\Delta \text{A}340/\text{min}}{0.0053}\times \frac{\text{Total volume}}{\text{Sample volume}}\;\times \;\text{dilution factor}.$$

The results were expressed in relation to the protein content of the samples (nmol min^−1^ mg^−1^ protein).

Catalase: The assay for the determination of CAT activity was based on Aebi [47]. In this assay, CAT activity is assessed by measuring the rate of removal of hydrogen peroxide (H_2_O_2_). The reaction can be followed by a decrease in absorbance as the H_2_O_2_ is converted into oxygen and water. At the end of the assay, H_2_O_2_ is consumed and CAT is inactivated. The total reaction volume of 3 mL was composed of 50 mM potassium phosphate buffer (pH 7.0) and 12.1 mM H_2_O_2_ as substrate. The reaction started by the addition of the samples into quartz cuvettes with an optical path length of 10 mm. The consumption of H_2_O_2_ [extinction coefficient of 0.04 ƐmM (mM^−1^ cm^−1^)] was monitored at 240 nm and 25°C, once every 15 s for a 180 s incubation period, using a Helios spectrophotometer (Unicam, UK). Standard CAT activity was measured using a bovine CAT solution (1523.6 U mL^−1^) as a positive control for the validation of the assay. CAT activity was calculated using the following equation:

$$\text{CAT activity }=\;\frac{\Delta \text{A}240/\text{min}}{0.04}\times \frac{\text{Total volume}}{\text{Sample volume}}.$$

The results were expressed in relation to the protein content of the samples (nmol min^−1^ mg^−1^ protein).

#### Lipid peroxidation

Lipid peroxidation was determined by the quantification of malondialdehyde (MDA), a specific end-product of the oxidative degradation process of lipids. The thiobarbituric acid reactive substances (TBARS) assay was used to quantify MDA as described by Rosa *et al*. [45]. Homogenates were treated with 8.1% sodium dodecyl sulfate, 20% trichloroacetic acid (pH 3.5), thiobarbituric acid and a 15:1 (v/v) mixture of n-butanol and pyridine. In the TBARS assay, the thiobarbituric acid reacts with the MDA to yield a fluorescent product, which was detected spectrophotometrically at 532 nm. MDA concentrations were calculated with the Microplate Manager 4.0 software (BIO-RAD, USA), based on an eight-point calibration curve (from 0 to 0.3 μM TBARS) using MDA bis (dimethyl acetal; Merck, Switzerland). The results were expressed in relation to the protein content of the samples (nmol mg^−1^ protein).

### Digestive enzymes

#### Preparation of tissue extracts

Two different groups of digestive enzymes were assayed: a) extracellular enzymes (more specifically, the pancreatic enzymes trypsin and amylase), and b) brush border enzymes linked to cell membranes (more specifically, the intestinal enzyme ALP).

Enzyme activities were measured in triplicates (using pooled larvae from each replicate tank) for each development stage (10, 20 and 30 dph larvae) under the different experimental conditions. Before homogenization, larvae were dissected in order to separate pancreatic and intestinal segments, as described by Cahu and Zambonino-Infante [48]. Samples were homogenized using a glass/PTFE Potter Elvehjem tissue grinder (Kartell, Italy) in 30 volumes (v/w) of ice-cold Tris-HCl (50 mM) and mannitol (2 mM) buffer at pH 7.0. The homogenates were then divided into two different aliquots of 1.5 mL and processed differently. Aliquots for assessing pancreatic enzymes were centrifuge at 3300 g (for 3 min) at 4°C, and the supernatants were removed for enzyme quantification. Intestinal brush border membranes for the determination of intestinal enzymes were purified according to Crane *et al*. [49]. Enzyme activities were expressed as specific enzyme activity, in units per milligram of protein (U mg^-1^ protein), and the soluble protein of crude enzyme extracts was quantified by the Bradford's method [44] using bovine serum albumin as standard.

#### Trypsin

Trypsin activity was assayed according to Holm *et al*. [50] using 0.1 MN α-benzoyl-DL-arginine p-nitroanilide (BAPNA) as substrate in 50 mM Tris-HCl buffer containing 20 mM CaCl_2_ at pH 8.2. The changes in absorbance were measured at 25°C during 2 min at 407 nm, using a UV-1800 Shimadzu UV spectrophotometer (Japan). One unit of trypsin activity corresponded to 1 μmol of 4-nitroaniline liberated in 1 min per mL of extracellular enzymatic extract, based on the extinction coefficient of the substrate [8200 ƐM (M^-1^ cm^-1^)].

#### Amylase

Amylase activity was quantified according to Metais [51] at 37°C and measured using soluble starch-iodine (0.3%) dissolved in Na_2_HPO_4_ buffer at pH 7.4 as substrate. Briefly, 50 μL of enzymatic extract was mixed with the substrate (3 g L^-1^ starch in Na_2_PO_4_, pH 7.4) and incubated for 30 min at 37°C. The reaction was stopped with 20 μL of 1 N HCL. After the addition of 2 mL of N/3000 iodine solution, the absorbance was read at 580 nm, using a UV-1800 Shimadzu UV spectrophotometer (Japan). One unit of α-amylase activity was defined as 1 mg of starch hydrolyzed per min and per mL of extracellular enzymatic extract at 37°C.

#### Alkaline phosphatase

ALP was quantified according to the procedure described by Bessey [52] and Hausamen [53] using 5 mM p-nitrophenyl phosphate (PNPP) as substrate in 30 mM Na_2_CO^3-^H_2_O and 1 mM MgCl^2-^6H_2_O buffer at pH 9.8. The enzymatic extract was mixed with the substrate solution and the change in absorbance was measured at 37°C during 2 min at 407 nm, using a UV-1800 Shimadzu UV spectrophotometer (Japan). One unit of ALP activity corresponded to 1 μmol of the substrate hydrolyzed in 1 min per mL of the brush border enzymatic extract (extinction coefficient of 18300 ƐM, M^-1^ cm^-1^).

### Statistical analyses

ANOVA was used to test whether significant differences existed between replicates of each experimental treatment. As no differences were found between replicates, all the samples from the same treatment were pooled and analyzed together. Three-way ANOVAs and Tukey HSD tests were then used to evaluate the effect of temperature, *p*CO_2_ and developmental stage on the metabolism (OCR), HSR (HSP70), antioxidant (GST and CAT), lipid peroxidation (MDA) and digestive enzyme (trypsin, amylase and ALP) activities.

Pearson’s correlation coefficients were used to analyze potential relationships between the variables analyzed in this study (OCR, HSR, lipid peroxidation, antioxidant and digestive enzymatic activities), and also with those obtained in our previous study with this species (namely survival, specific growth rates and skeletal deformities; see [29]).

All statistical analyses were performed for a significance level of 0.05, using Statistica 12.0 software (StatSoft Inc., Tulsa, USA).

## Results

### Oxygen consumption rates

**Fig 1:**
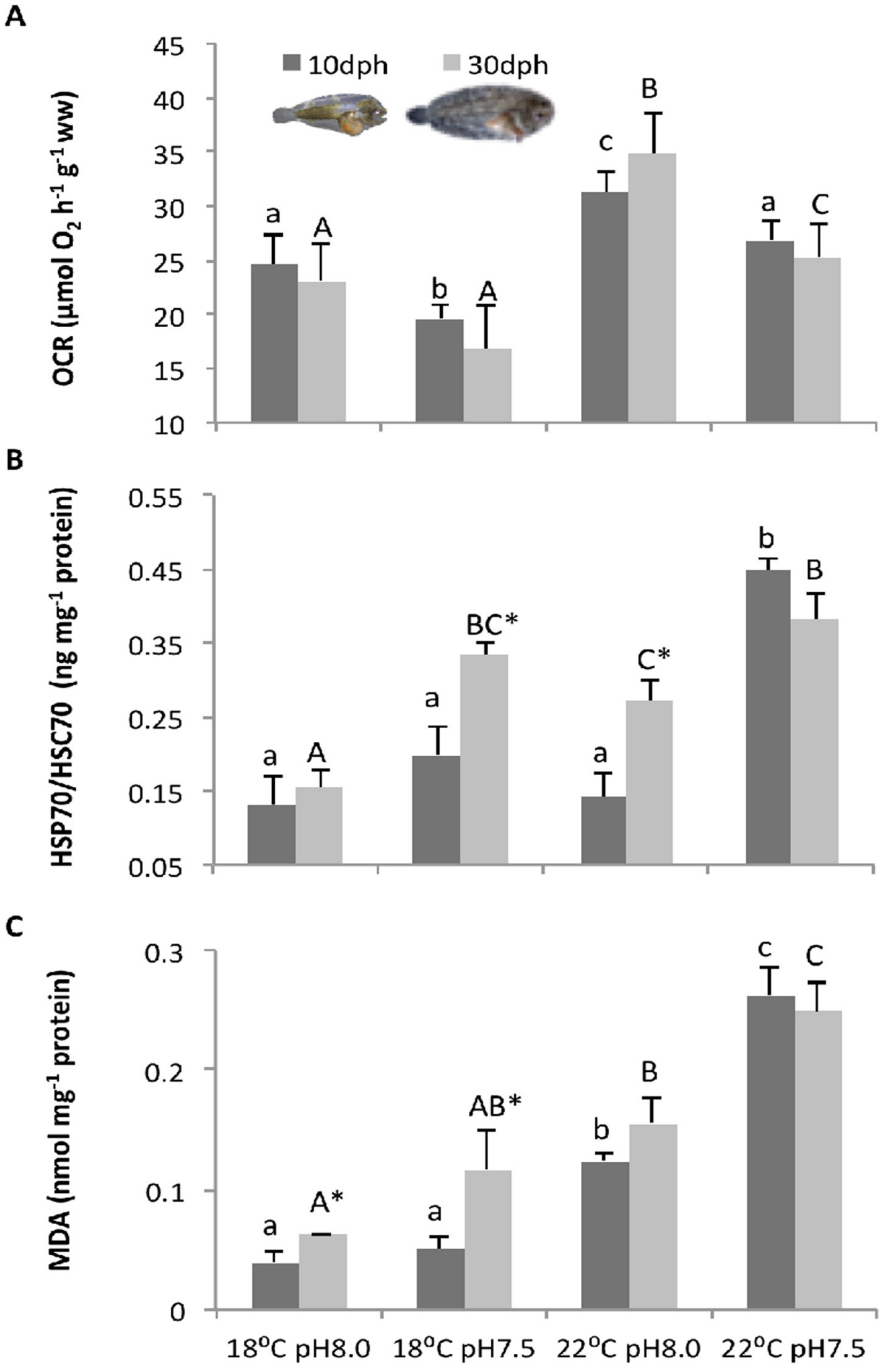
Impact of ocean acidification and warming on the metabolism, heat shock response and lipid peroxidation of *Solea senegalensis* larvae. **A**) Oxygen consumption rates (OCR), **B**) heat shock protein 70 (HSP70) concentrations, and **C**) malondialdehyde (MDA) levels in 10 and 30 dph larvae at different temperature and pH scenarios. Values are given as means + SD. Different letters (lower case for 10 dph larvae; capital letters for 30 dph) represent significant differences between the different climate scenarios (p<0.05). Asterisks represent significant differences between 10 and 30 dph larvae for the same treatment (p<0.05).

The effect of warming and high *p*CO_2_ on the metabolic rates of *S*. *senegalensis* larvae is presented in Fig 1A (see also S2 Table). OCR were significantly affected by temperature and *p*CO_2_ (p<0.05), but not by developmental stage (p>0.05). Sole larvae displayed significantly higher OCR under normocapnia (23.11 μmol O_2_ h^-1^ g^-1^ at present-day temperature and 34.85 μmol O_2_ h^-1^ g^-1^ at the future warming scenario). At higher *p*CO_2_, OCR decreased significantly to 16.82 and 25.28 μmol O_2_ h^-1^ g^-1^ (at present-day temperature and future warming scenario, respectively). No significant interaction was found between the three factors (p>0.05).

### Heat shock response

The HSR of sole larvae was significantly (p<0.05) affected by temperature and *p*CO_2_, and also by developmental stage (Fig 1B; see also S2 Table). Additionally, a significant interaction was observed between these three factors (p<0.05). The HSR (inducible HSP70) increased under hypercapnia in both pre- and post-metamorphic larval stages, especially under the warming treatment. In general, post-metamorphic larvae presented a stronger HSR than pre-metamorphic larvae (16.7 to 92.9 percentage points higher), except under the warming and high *p*CO_2_ scenario, where HSR decreased 17.9 percentage points and the differences between stages were not statistically significant.

### Antioxidant enzymes

**Fig 2:**
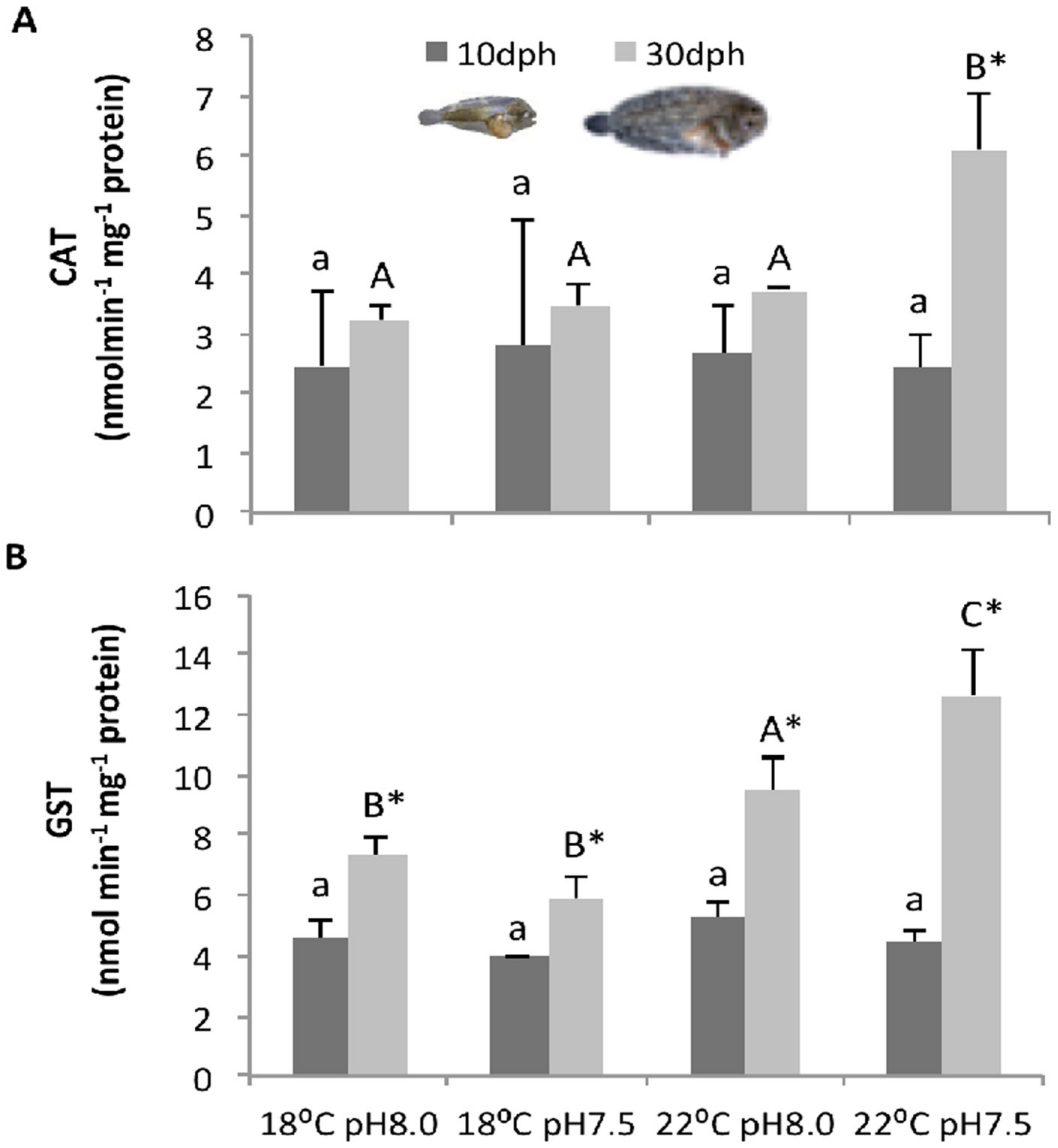
Impact of ocean acidification and warming on the antioxidant response of *Solea senegalensis* larvae. **A)** catalase (CAT), and **B)** glutathione S-transferase (GST) activities of 10 and 30 dph larvae at different temperature and pH scenarios. Values are given as means + SD. Different letters (lower case for 10 dph larvae; capital letters for 30 dph) represent significant differences between the different climate scenarios (p<0.05). Asterisks represent significant differences between 10 and 30 dph larvae for the same treatment (p<0.05).

The impact of high *p*CO_2_ and environmental warming on antioxidant enzymes (CAT and GST) of *S*. *senegalensis* larvae is shown in Fig 2 (see also S2 Table).

CAT activity (Fig 2A) was significantly affected by developmental stage (p<0.05), but not by temperature and *p*CO_2_ or by the interaction between factors (p>0.05). The highest value of CAT activity (6.10 ± 0.95 nmol min^-1^ mg^-1^ protein) was observed in the post-metamorphic larvae exposed to warming and high *p*CO_2_. Pre-metamorphic larvae showed always lower values than post-metamorphic larvae, and no significant variation (p>0.05) was observed among treatments (between 2.42 ± 0.67 and 2.81 ± 1.43 nmol min^-1^ mg^-1^ protein).

GST activity (Fig 2B) was significantly affected by temperature and developmental stage, as well as by the interactions between factors (p<0.05). GST activity in pre-metamorphic larvae was also lower than in post-metamorphic larvae (p<0.05), and similar in all treatments (p>0.05). In contrast, the GST activity of post-metamorphic larvae increased significantly with temperature (p<0.05). The highest value (12.64 ± 1.51 nmol min^-1^ mg^-1^ protein) was observed under the combined effect of warming and high *p*CO_2_.

### Lipid peroxidation

Lipid peroxidation (based on MDA levels) was also significantly affected by temperature, *p*CO_2_, developmental stage, and the interaction between these three factors (p<0.05) (Fig 1C, see also S2 Table). Lipid peroxidation increased significantly with warming in both developmental stages. The lowest value (0.039 ± 0.007 nmol mg^-1^ protein) was found in pre-metamorphic larvae exposed to the present-day conditions. The effect of ocean acidification on MDA levels was only significantly noted under the warming scenario. In fact, the highest MDA values (0.26 ± 0.02 and 0.25 ± 0.03 nmol mg^-1^ protein in pre- and post-metamorphic larvae, respectively) were found when larvae were exposed to the combined effects of higher temperature and *p*CO_2_. MDA buildup was generally more pronounced in post-metamorphic larvae, except under the future combined scenario.

### Digestive enzymes

**Fig 3:**
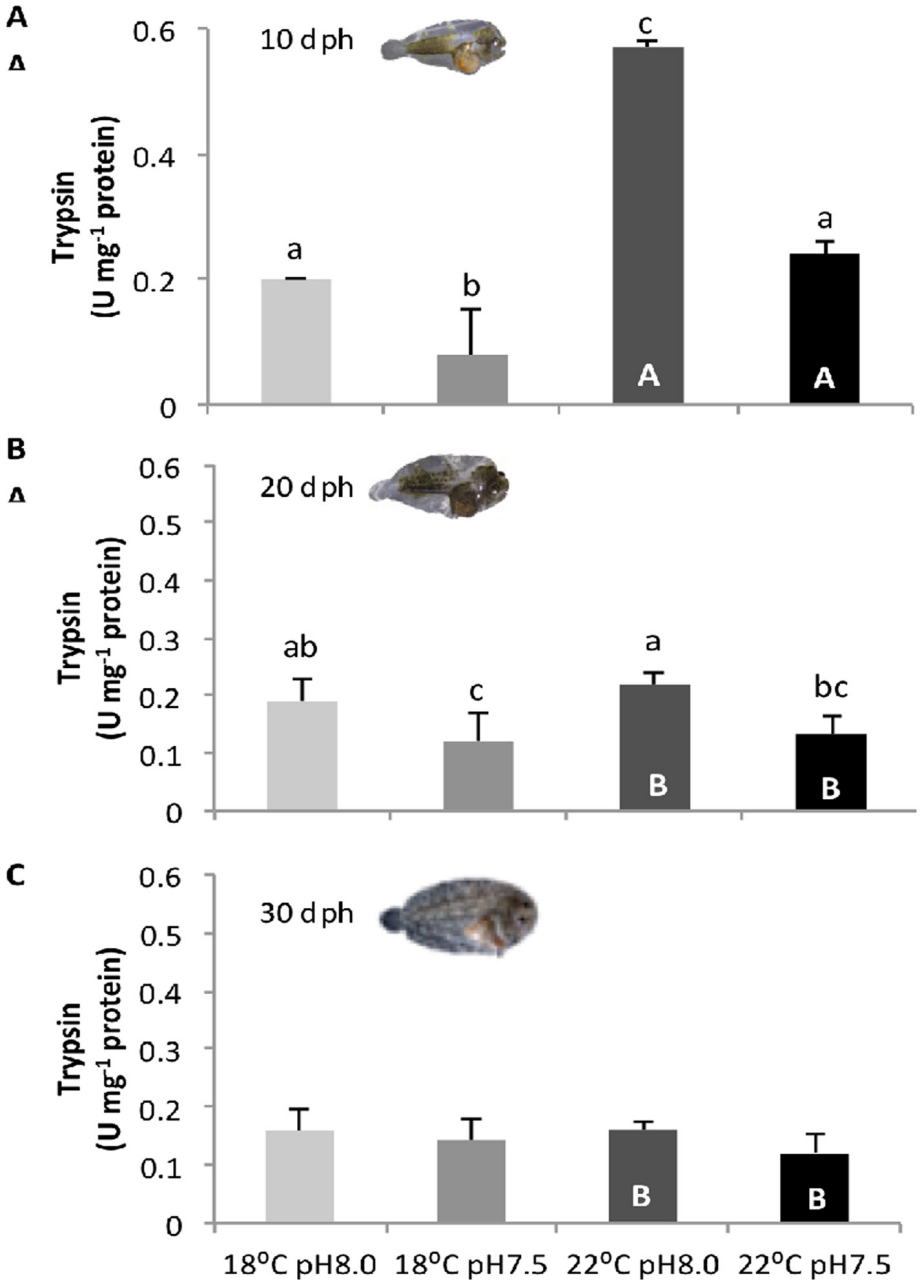
Impact of ocean acidification and warming on the trypsin activity of *Solea senegalensis* larvae. Enzyme activity in **A)** 10 dph, **B)** 20 dph, and **C)** 30 dph larvae at different temperature and pH conditions. Values are given as means + SD. Different letters represent significant differences between the different climate scenarios (p<0.05). Lower-case letters indicate differences between treatments at the same development stage; capital letters represent differences between 10, 20 and 30 dph larvae for the same treatment.

**Fig 4:**
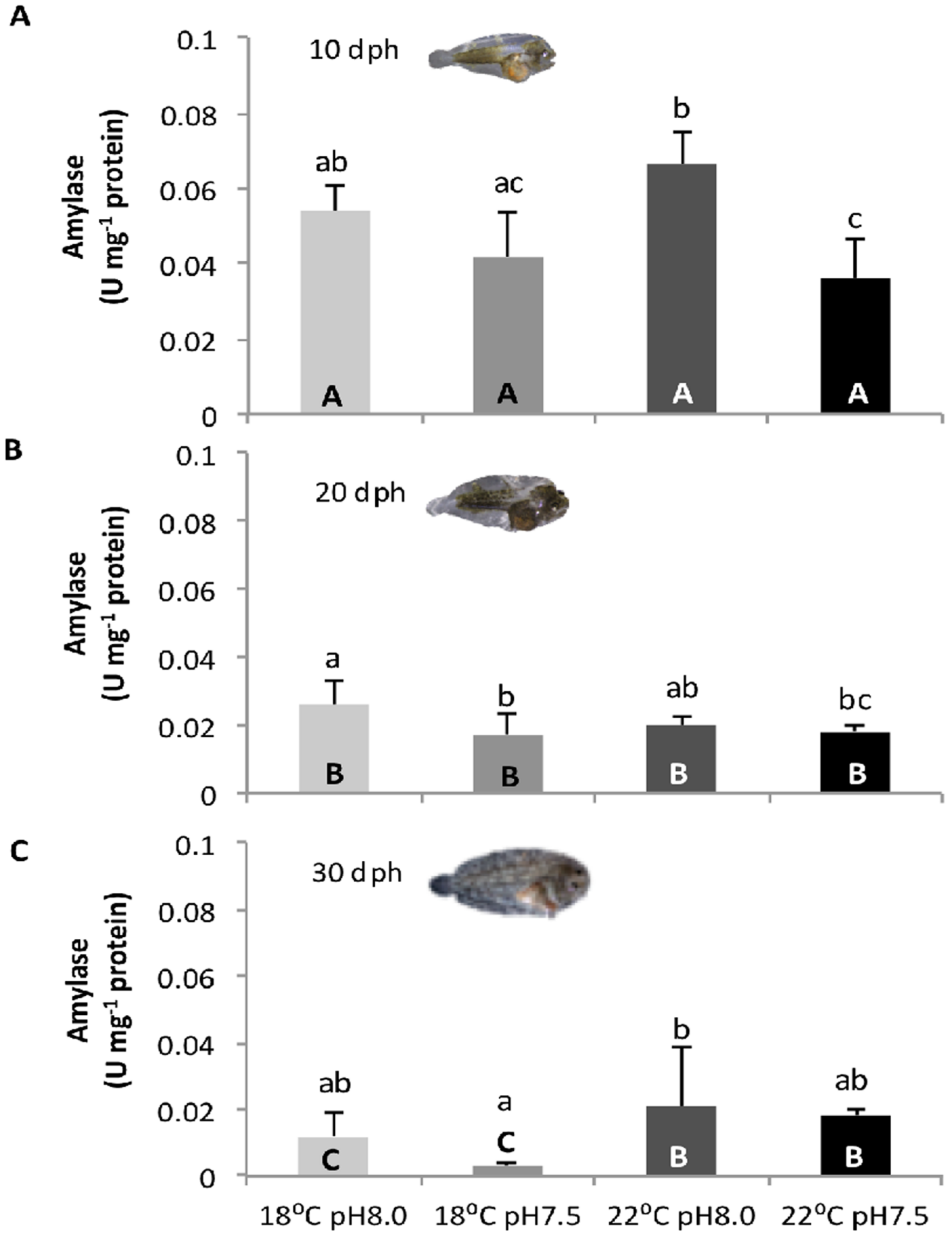
Impact of ocean acidification and warming on the amylase activity of *Solea senegalensis* larvae. Enzyme activity in **A)** 10 dph, **B)** 20 dph, and **C)** 30 dph larvae at different temperature and pH conditions. Values are given in mean + SD. Different letters represent significant differences between the different climate scenarios (p<0.05). Lower-case letters indicate differences between treatments at the same development stage; capital letters represent differences between 10, 20 and 30 dph larvae for the same treatment.

**Fig 5:**
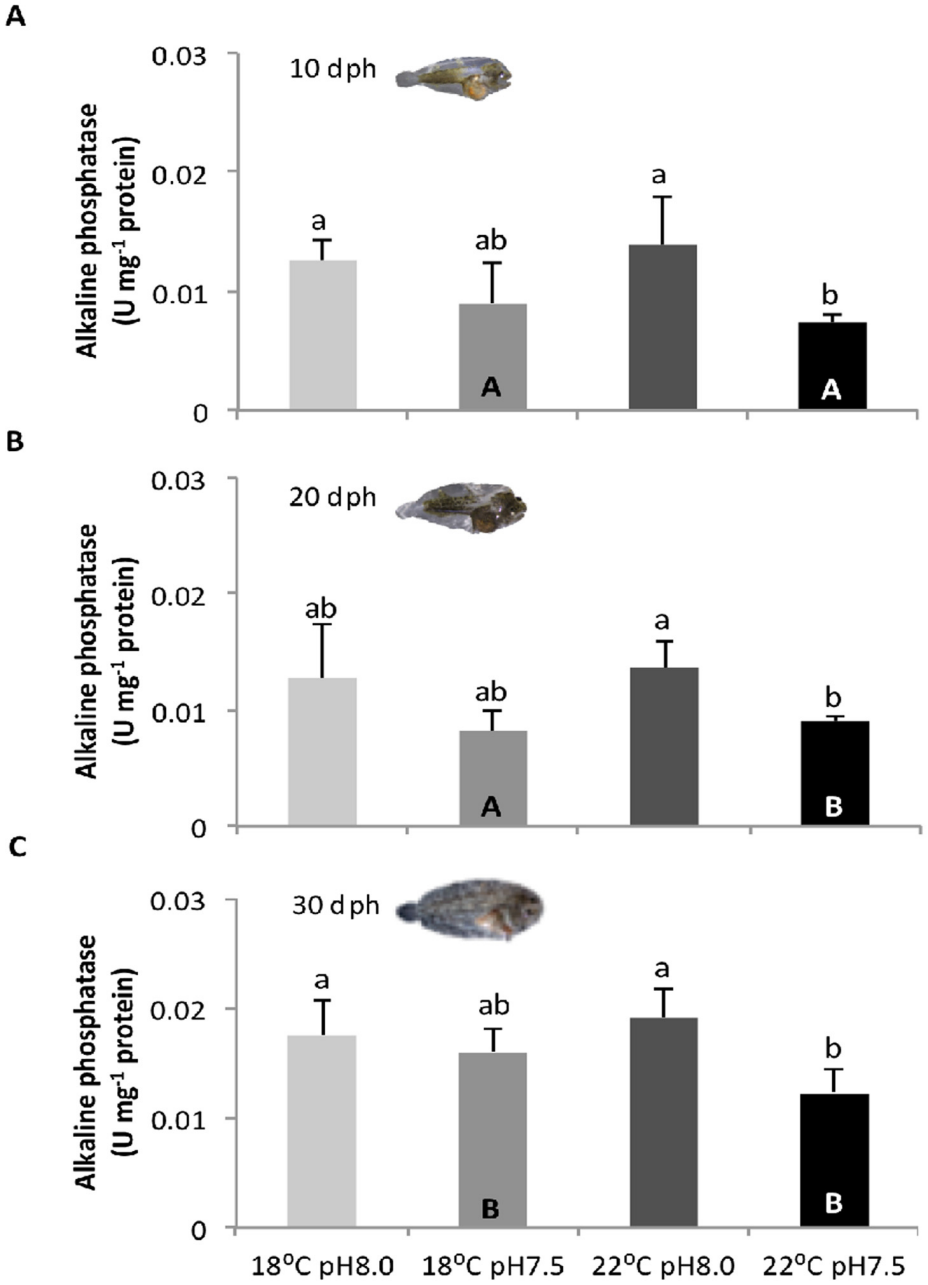
Impact of ocean acidification and warming on the alkaline phosphatase activity of *Solea senegalensis* larvae. Enzyme activity in **A)** 10 dph, **B)** 20 dph, and **C)** 30 dph larvae at different temperature and pH conditions. Values are given in mean + SD. Different letters represent significant differences between the different climate scenarios (p<0.05). Lower-case letters indicate differences between treatments at the same development stage; capital letters represent differences between 10, 20 and 30 dph larvae for the same treatment.

The effect of warming and high *p*CO_2_ on digestive enzymes of sole larvae is presented in Figs 3–5 (see also S2 Table). Both extracellular enzymes (trypsin and amylase) increased throughout development, while the brush border enzyme ALP significantly increased.

Trypsin activity (Fig 3) was significantly affected by temperature, *p*CO_2_ and developmental stage, as well as by the interactions between factors (p<0.05). Trypsin activity increased with temperature only in 10 dph larvae. Regardless of temperature, trypsin activity decreased significantly with hypercapnia in both 10 and 20 dph larvae (p<0.05), but not in 30 dph larvae (p>0.05). The highest trypsin activity (0.57 ± 0.02 U mg^-1^ protein) was observed in 10 dph larvae under warming and normocapnia, and the lowest value (0.08 ± 0.01 U mg^-1^ protein) was observed under present-day temperature and hypercapnic conditions.

Amylase activity (Fig 4) was also significantly affected by the three factors (temperature, *p*CO_2_ and developmental stage), as well as by most interactions between them (p<0.05). Amylase activity was also highest (0.07 ± 0.01 U mg^-1^ protein) in 10 dph larvae under warming and normocapnia. Before metamorphosis, amylase activity decreased significantly (p<0.05) with warming and hypercapnia (up to 0.036 ± 0.011 U mg^-1^ protein), but showed no significant variation (p>0.05) at 20 dph (values between 0.018 ± 0.002 and 0.026 ± 0.007 U mg^-1^ protein) and 30 dph (values between 0.003 ± 0.001 and 0.018 ± 0.002 U mg^-1^ protein).

ALP activity (Fig 5) was significantly affected by *p*CO_2_ and development stage (p<0.05), but not by temperature neither by the interaction of the three factors (p>0.05). ALP activity decreased with hypercapnia, especially when combined with warming (p<0.05). The lowest activity level of ALP (0.007 U mg^-1^ protein) was detected at 10 dph under warming and hypercapnic exposure, while the highest value (0.019 U mg^-1^ protein) was detected at 30 dph under warming and normocapnic conditions.

### Correlation between variables

The correlations between the variables analyzed in the present study for 10 and 30 dph larvae are presented in Tables 1 and 2, respectively. Table 2 also includes the correlations between the variables analyzed in the present study with those obtained in our previous study [29].

**Table 1:** Correlation analysis between physiological and biochemical variables of 10 dph *Solea senegalensis* larvae.

	OCR	HSP	MDA	CAT	GST	Trypsin	Amylase
HSP	0.03						
MDA	0.47	0.89					
CAT	-0.37	-0.46	-0.51				
GST	0.95	-0.29	0.17	-0.18			
Trypsin	0.94	-0.22	0.25	-0.05	0.98*		
Amylase	0.61	-0.77	-0.40	0.16	0.83	0.78	
ALP	0.50	-0.84	-0.52	0.09	0.74	0.65	0.98*

Pearson’s coefficients between the variables analyzed in the present study, namely oxygen consumption rates (OCR), heat shock protein (HSP) concentrations, malondialdehyde (MDA) levels, antioxidant enzyme activities (catalase—CAT and glutathione S-transferase—GST) and digestive enzyme activities (trypsin, amylase and alkaline phosphatase—ALP). Asterisks represent significant correlations (p<0.05).

**Table 2:** Correlation analysis between physiological, biochemical and morphological variables of 30 dph *Solea senegalensis* larvae.

	Survival	SGR	Malformations	OCR	HSP	MDA	CAT	GST	Trypsin	Amylase
SGR	-0.27									
Malformations	-0.96*	0.05								
OCR	-0.16	0.99*	-0.03							
HSP	-0.93*	-0.03	0.99*	-0.12						
MDA	-0.98*	0.42	0.88	0.30	0.83					
CAT	-0.88	0.28	0.76	0.13	0.71	0.94*				
GST	-0.76	0.65	0.55	0.52	0.47	0.88	0.90*			
Trypsin	0.83	0.17	-0.81	0.31	-0.81	-0.80	-0.90	-0.62		
Amylase	-0.37	0.97*	0.13	0.92*	0.03	0.54	0.47	0.80	-0.03	
ALP	0.70	0.27	-0.69	0.41	-0.69	-0.69	-0.85	-0.55	0.98*	0.05

Pearson’s coefficients between the variables analyzed in the present study, namely oxygen consumption rates (OCR), heat shock protein (HSP) concentrations, malondialdehyde (MDA) levels, antioxidant enzyme activities (catalase—CAT and glutathione S-transferase—GST) and digestive enzyme activities (trypsin, amylase and alkaline phosphatase—ALP), and those obtained in our previous study with this species [29], namely survival, specific growth rates (SGR) and the incidence of skeletal malformations. Asterisks represent significant correlations (p<0.05).

The metabolism of 10 dph larvae was positively correlated with GST (r = 0.95; p = 0.041) and trypsin (r = 0.94; p = 0.044), while the metabolism of 30 dph larvae was found to be positively correlated with amylase (r = 0.92; p = 0.040). Moreover, based on our previous findings [29], we found that the incidence of skeletal deformities in 30 dph larvae was positively correlated with HSP levels (r = 0.99; p = 0.005), while specific growth rates (SGR) were positively correlated with OCR (r = 0.99; p = 0.014) and amylase levels (r = 0.97; p = 0.030). On the other hand, survival was negatively correlated with HSP (r = -0.93; p = 0.049) and MDA levels (r = -0.98; p = 0.025). No other significant relationship was found (p>0.05).

## Discussion

Early life stages of marine fish are expected to be particularly sensitive to environmental stressors, due to the lack or low functional capacity of some organ systems (e.g., gill epithelium) and to the high rates of metabolism needed to fuel growth and development. In our previous study with *S*. *senegalensis* eggs and larvae [29], the exposure to future conditions caused a decline in the hatching success, larval survival and growth of this flatfish species. Moreover, hypercapnia and warming amplified the incidence of skeletal deformities (by 32%), including severe deformities such as lordosis, scoliosis and kyphosis. Here we show that these climate change-related variables also affected the metabolism, HSR, lipid peroxidation, as well as the activity of antioxidant and digestive enzymes.

The metabolic rate of *S*. *senegalensis* larvae increased with temperature as expected (following normal Q_10_ values), but exposure to hypercapnic conditions triggered a 25% reduction in OCR. Metabolic depression, and the consequent reduction of total energy expenditure, is an important strategy to survive under acute environmental stress [54,55], because it allows organisms to put some biological processes in stand-by as a strategy for saving energy, prioritizing the survival of the individual [2,56]. Protein synthesis is an ATP-consuming process, and a reduced ATP demand of most cells might lead to a reduction in protein synthesis, which would by definition restrict growth [57,58]. Indeed, the lower OCR in sole larvae was strongly and positively correlated with lower SGR.

Most organisms display an integrated stress response (heat shock response and antioxidant enzyme activity) to prevent the increase in ROS formation [59] and the protein damage and unfolding [60] caused by environmental stressful conditions. The ability of elevated cellular HSP levels to strengthen thermal and chemical tolerance in animals is well documented [61–63]. In the present study, the exposure of sole larvae to warmer temperatures and higher *p*CO_2_ levels triggered an increase in HSP70 levels in both developmental stages, thus indicating a stress response. Marine organisms possess also a powerful set of antioxidant enzymes that helps to detoxify ROS and reduce the negative effects on fitness [64,65]. Indeed, CAT and GST concentrations of post-metamorphic larvae increased by 88 and 72%, respectively, from present-day to forthcoming conditions. However, pre-metamorphic larvae may lack a fully developed antioxidant defense system and may be more exposed to tissue damage, as there were no differences in CAT and GST concentrations between treatments. Altogether, inducible HSP70, CAT and GST responses seem to constitute an integrated response of post-metamorphic larvae during exposure to warmer temperatures and hypercapnic conditions.

Despite the increment of HSR and antioxidant enzyme activities, this significant up-regulation was not effective against cellular injuries. Lipid peroxidation still increased under high temperature and *p*CO_2_ conditions, as suggested by the higher MDA levels, a specific end-product of the oxidative degradation process of lipids. Environmental factors are known to be responsible for significant changes in MDA levels indicating that organisms are facing some adjustments due to oxidative stress conditions. In addition to the effect of temperature, high *p*CO_2_ was further responsible for exacerbating the heat-induced cellular injuries. This matches findings in crustaceans that show an earlier onset of thermal limitation under elevated *p*CO_2_ as a general principle [1,66,67].

Besides affecting the stress response (HSR and oxidative stress tolerance) of sole larvae, future ocean conditions also affected the activity of digestive enzymes. The ontogenetic development of the digestive system of sole larvae occurred as expected [16], characterized by a decrease in the activity of pancreatic enzymes followed by an increase in intestinal (brush border) enzyme activity. These opposing trends of ontogenetic variation may suggest the maturation of enterocytes, but further histological analysis would be necessary to confirm it. Regardless of this, elevated CO_2_ conditions led to a general decrease in the activity of the digestive enzymes, both pancreatic and intestinal enzymes, especially in pre-metamorphic sole larvae. Morphological and physiological impairments in the digestive system (namely gut and pancreas) of fish early life stages under ocean acidification have already been observed [27,28], but no connection has been established between altered functional development and digestive enzymatic activities.

All together, the results from the present study indicate that ocean warming and acidification pose significant stress to *S*. *senegalensis* larvae, especially to pre-metamorphic stages. Besides affecting the metabolism, HSP and antioxidant responses, lipid peroxidation and the activity of digestive enzymes, the impact of these climate change-related variables on some of these physiological and biochemical variables was further translated into fish performance. As mentioned above, lower oxygen consumption rates under hypercapnia were correlated with reduced larval growth. Moreover, the increase in HSP and MDA levels under high temperature and *p*CO_2_ conditions, which are indicators of stress and tissue damage, was negatively correlated with larval survival. HSP levels were also positively correlated with the incidence of skeletal deformities. Other studies have shown that conditions that induce the heat shock response may also induce abnormal development [68–70]. In fact, environmental stress factors are among the most important factors that can induce skeletal deformities during fish development [71]. More studies should establish links between biochemical markers, physiological and morphological parameters in an attempt to demonstrate the effects from cellular processes up to the whole-animal level, in order to provide a more conclusive evidence of the sensitivity of marine fish early life stages to ocean climate change.

## Supporting Information

S1 Table Seawater carbonate chemistry data for the different climate change scenarios. Total carbon (C_T_), carbon dioxide partial pressure (*p*CO_2_), bicarbonate concentration (HCO_3_^-^) and aragonite saturation state of seawater (Ω_arag_) were calculated with CO2SYS using salinity, temperature, pH and total alkalinity (A_T_). Values are means ± SD.
(PDF)

S2 Table ANOVA results. Results of three-way ANOVA evaluating the effect of temperature, *p*CO_2_ and development stage on the oxygen consumption rate (OCR), heat shock proteins (HSP), lipid peroxidation (MDA—malondialdehyde), antioxidant enzymes (GST—Glutathione S-transferase, and CAT—catalase) and digestive enzymes (trypsin, amylase, and ALP—alkaline phosphatase) of *Solea senegalensis* larvae under the effect of ocean warming and acidification.
(PDF)
