# Supplementary material for: Oxidative Stress and Digestive Enzyme Activity of Flatfish Larvae in a Changing Ocean
Source: PLoS One. 2015 Jul 29;10(7):e0134082. doi: 10.1371/journal.pone.0134082 (PMC4519323; doi:10.1371/journal.pone.0134082)
Supplement: S1 Table — Total carbon (CT), carbon dioxide partial pressure (pCO2), bicarbonate concentration (HCO3 -) and aragonite saturation state of seawater (Ωarag) were calculated with CO2SYS using salinity, temperature, pH and total alkalinity (AT). Values are means ± SD. (PDF) [file pone.0134082.s001.pdf]

Supporting Information

S1 Table. Seawater carbonate chemistry data for the different climate change scenarios.

Temperature (ºC)	pH (Total scale)	A_T [µmol kg^{-1}SW]	C_T [µmol/kg^{-1} SW]	pCO_2 [µatm]	HCO_3^- [µmol kg^{-1}]	Ω_{arag}
22.02 ± 0.42	8.03 ± 0.05	2335.74 ± 89.09	2148.20 ± 81.43	424.53 ± 19.97	1985.25 ± 75.28	2.24 ± 0.08
22.12 ± 1.01	7.51 ± 0.05	2317.40 ± 36.40	2314.73 ± 36.72	1654.20 ± 49.06	2194.88 ± 34.84	0.78 ± 0.01
18.20 ± 0.40	8.02 ± 0.04	2305.70 ± 80.54	2141.80 ± 76.78	400.00 ± 66.71	1993.35 ± 72.21	1.95 ± 0.07
18.15 ± 0.29	7.50 ± 0.03	2281.07 ± 61.89	2290.90 ± 62.73	1607.90 ± 24.78	2173.55 ± 59.50	0.67 ± 0.02
