# Supplementary material for: Oxidative Stress and Digestive Enzyme Activity of Flatfish Larvae in a Changing Ocean
Source: PLoS One. 2015 Jul 29;10(7):e0134082. doi: 10.1371/journal.pone.0134082 (PMC4519323; doi:10.1371/journal.pone.0134082)
Supplement: S2 Table — Results of three-way ANOVA evaluating the effect of temperature, pCO2 and development stage on the oxygen consumption rate (OCR), heat shock proteins (HSP), lipid peroxidation (MDA—malondialdehyde), antioxidant enzymes (GST—Glutathione S-transferase, and CAT—catalase) and digestive enzymes (trypsin, amylase, and ALP—alkaline phosphatase) of Solea senegalensis larvae under the effect of ocean warming and acidification. (PDF) [file pone.0134082.s002.pdf]

Supporting Information

S2 Table. ANOVA results.

Measure	Factor	df	F	p
OCR	Temperature (*T*)	1	111.8	0.000
	pH	1	61.5	0.000
	Stage	1	0.5	0.504
	T x pH	1	0.7	0.399
	T x stage	1	3.7	0.060
	pH x stage	1	4.2	0.046
	T x pH x stage	1	1.5	0.222
	Error	50		
HSP	Temperature (*T*)	1	63.0	0.000
	pH	1	138.9	0.000
	Stage	1	14.9	0.003
	T x pH	1	11.1	0.008
	T x stage	1	1.8	0.209
	pH x stage	1	2.6	0.139
	T x pH x stage	1	27.5	0.000
	Error	10		
MDA	Temperature (*T*)	1	190.5	0.000
	pH	1	61.0	0.000
	Stage	1	8.1	0.019
	T x pH	1	19.5	0.002

	T x stage	1	3.6	0.090
	pH x stage	1	0.0	0.959
	T x pH x stage	1	5.5	0.044
	Error	9		
GST	Temperature (*T*)	1	63.8	0.000
	pH	1	0.0	0.941
	Stage	1	184.0	0.000
	T x pH	1	13.1	0.003
	T x stage	1	41.8	0.000
	pH x stage	1	7.9	0.015
	T x pH x stage	1	13.1	0.003
	Error	13		
CAT	Temperature (*T*)	1	3.9	0.084
	pH	1	3.3	0.109
	Stage	1	17.2	0.003
	T x pH	1	1.1	0.334
	T x stage	1	4.8	0.058
	pH x stage	1	2.8	0.134
	T x pH x stage	1	3.2	0.111
	Error	8		
Trypsin	Temperature (*T*)	1	85.3	0.000
	pH	1	125.1	0.000
	Stage	2	61.1	0.000
	T x pH	1	18.1	0.000
	T x stage	2	75.2	0.000
	pH x stage	2	33.6	0.000
	T x pH x stage	2	9.8	0.000

	Error	43		
Amylase	Temperature (*T*)	1	4.6	0.035
	pH	1	32.1	0.000
	Stage	2	122.3	0.000
	T x pH	1	0.1	0.719
	T x stage	2	4.5	0.014
	pH x stage	2	7.3	0.001
	T x pH x stage	2	4.7	0.012
	Error			
ALP	Temperature (*T*)	1	0.8	0.362
	pH	1	56.3	0.000
	Stage	2	36.9	0.000
	T x pH	1	7.6	0.007
	T x stage	2	0.5	0.588
	pH x stage	2	0.8	0.472
	T x pH x stage	2	1.0	0.358
	Error			
